# Effects of fine particulate matter air pollution on survival of *Heliconius ethilla* (Godart, 1819)

**DOI:** 10.1038/s41598-024-78347-w

**Published:** 2024-11-29

**Authors:** A. M. M. Charpinel, J. C. Féres, F. C. C. Barreto

**Affiliations:** Departamento de Ciências Biológicas, Universidade Federal do Espírito Santo, Vitória, ES Brazil

**Keywords:** Urbanization, Air pollution, Lepidoptera, Life table, Mortality, Viable populations, Urban ecology, Ecology, Conservation biology

## Abstract

Human activities affect natural ecosystems worldwide and can generate negative effects on insect species such as growth inhibition, developmental abnormalities and reduction of reproductive and survival rates. Our study focused on fine particulate matter, a pollutant known to cause mechanical obstruction, heavy metal intoxication, and stress in Lepidoptera larvae. *Heliconius ethilla* (Lepidoptera: Nymphalidae) is a Heliconiinae found in the southeastern region of Brazil. We started our study with 3255 eggs, from which 69 stage 3 larvae were randomly separated as the treatment group. The larvae were fed with Passiflora edulis leaves and the SPM was added before being offered to the treatment group in an increasing concentration according to the age of the larvae and the leaf, simulating the gradual deposition of SPM in the environment. Our results demonstrate that Sedimentable Particulate Matter negatively impacted mortality rates, pupal weight, and body size. The results strongly indicate that the presence of SPM negatively impacts the survival, development and potentially the reproductive success of H. ethilla. A reduction in the population size and a consequent decrease in the chances of long-term survival of the species can be expected.

## Introduction

Human interventions has been affecting the natural ecosystems all over the world^1^. Modern agricultural practices, industrialization and increased vehicular use have led to high concentrations of pollutants in the environment^2^. These pollutants are regularly getting, for example, into air and soil^3^. Among the air pollutants, the most common are the ones called the six criteria pollutants. These air pollutants are the particulate matter (PM), ozone ($O_3$), carbon monoxide (CO), nitrogen oxides ($NO_x$), sulfur dioxide ($SO_2$) and lead (Pb). The Clean Air Act requires the U.S Environmental Protection Agency (U.S EPA) to set National Ambient Air Quality Standards (NAAQS) for these pollutants^4^ and in European Union, the Ambient Air Quality Directives (AAQD) set air quality standards for 12 air pollutants^5^, including these six criteria air pollutants. The World Health Organization (WHO) creates guidelines based on the health hazards reviews of the particulate matter (PM), ozone ($O_3$), carbon monoxide (CO), nitrogen oxides ($NO_x$) and sulfur dioxide ($SO_2$)^6^.

Total Suspended Particles (TSP) also known as particulate matter air pollution, are airborne particulates represented by all the particles suspended in the atmosphere with a large particle size range and capable of being sampled^7^. Typically, the aerodynamic diameter of these particles ranges from $<0.1 \mu$m to greater than 100 μm^8^. These particles are grouped in coarse, fine and ultrafine particles. The $PM_{10}$ are airborne particles with an aerodynamic diameter of less than 10 μm that penetrate the respiratory system and includes those inhalable particles that are sufficiently small to penetrate to the thoracic region and are mainly emitted by mechanical processes in construction activities and by the resuspension of particles in roadways due to traffic or wind erosion, among others. The $PM_{10}$ includes the airborne particles that range from 2.5 $\mu$m to 10 μm and are known as coarse particles ($PM_{2.5}$-$PM_{10}$). The $PM_{2.5}$ are the fine particles in suspension that have aerodynamic diameter of less than 2.5 $\mu$m and are produced mainly in combustion processes^6,7,9^. The $PM_{2.5}$ are stored in the bronchioles, while the other fractions of particles smaller than 10 μm are retained in the nose and nasopharynx, and can later be eliminated from the respiratory system by the defense mechanisms of the human organism^7^. The $PM_{0.1}$ have an aerodynamic diameter of less than 0.1 μm^6,9,10^ and are also known as nanoparticles (NPs) and ultrafine particles (UFPs)^11^. The ultrafine particles can not only penetrate the alveoli and be deposited in the lungs, but could also be transfered to other organs^12,13^. Due to its size, highly chemical reactivity and rapid changes it is difficult to determine its source of emissions^14,15^, mainly because there is a high instrumental and methodological demand for the detection^16^ and absence of evaluation standards and regular monitoring^17^. The $PM_{0.1}$ health effects are also poorly understood because of the lack of long-term exposure studies^18^ and assessment metrics^17^.

Research on the impact of air pollution on health in Latin American countries yield results similar to those in other locations in the world^19^. As for health outcomes, respiratory and cardiovascular diseases are the ones most commonly associated with air pollution^6,9^. The World Health Organization (WHO) released estimates in an update in 2018 with the data from 2016 reporting that there were 4.2 million deaths associated to the burden of diseases from ambient air pollution, from which 38% of the deaths were because of Ischemic Hearth Disease (IHD), 20% were due to stroke, 18% were because of Chronic Obstructive Pulmonary Disease (COPD), 18% due to Acute lower respiratory disease (ALRI) and 6% of deaths were associated to lung cancer^20^. According to a global burden of disease analysis, the deaths attributed to air pollution was the fourth largest cause of death for both males and females in 2019, representing 6.67 million deaths and it was also responsible for approximately 200 million disability-adjusted life years (DALYs)^21^.

Air pollution is an almost ubiquitous component of urban environments, which were defined by Ehler (1978) as a wide shared connection that reflects the enormous variety of ecological situations at cities, towns and their surrounding areas that collectively encompass numerous continua of disturbance and change. Urban areas are commonly known as “green spaces” or “open spaces”. Whitmore and collaborators (2002) define urban open spaces as any vegetated areas (green areas) which includes nature reserves, private and public gardens, sport and recreational grounds, roadsides, rail verges and transmission line servitudes, cultivated, derelict and undeveloped land. Urbanization is associated with habitat degradation, including decreased plant species diversity, reduced water quality, and increased air and soil pollution^22^. Researches on the impact of urbanization on biodiversity have focused primarily on vertebrates, including reptiles^23^, amphibians^24^, mammals^25^ and birds^26,27^. However, there has been less emphasis on understanding the consequences of habitat loss and fragmentation on terrestrial invertebrates^28,29^.

Insects have strong relationship with ecology^30^ and are typically the overwhelmingly dominant invertebrate faunal group^31^. They have been used popularly and extensively in biomonitoring and bioassessment programs throughout the world^30,31^. More than 17000 species of butterfly are found worldwide^32^. The butterflies have ecological fidelity and are sensitive to environmental changes and to the quality of the environment, and play a crucial role in indicating environmental quality. Ramírez-Restrepo and MacGregor-Fors (2017) reported negative effects of urbanization on butterflies based on reviewed studies. Heidari and collaborators (2022) found out that the insect herbivore diversity was negatively associated with the CO concentration. MacGregor-Fors and collaborators (2015) demonstrated that butterflies were the most sensitive wildlife group to urbanization and Matteson and collaborators (2013) found a lower diversity (species richness and abundance) of flower-visiting insects, including butterflies, in heavily developed neighborhoods compared to urban green areas. Butterflies respond differently to urbanization depending on their area of distribution and taxonomic identity^33,34^. Specialist butterflies decreases with increasing urbanization^33,35–37^ and urbanization can lead to local extinctions of infrequent, not abundant, specialist butterfly species^33^.

The group Lepidoptera are also used as environmental indicators of heavy metals and carbon dioxide concentration in locations close to industrial and within urban areas^38^. Chen and collaborators (2005) highlight the successful use of these insects for environmental pollution and heavy metal contamination assessment, both near industrial zones and within urban areas. Acute and chronic effects of air pollution on some insects are frequently reported in the form of growth inhibition, developmental abnormalities, reduced reproduction and decreased hatchability^39^. For example, on mulberry leaves, oil-soaked lesions induced by sulfur dioxide were associated with reduced feeding rate, inactivity, non-uniform growth, delayed cocooning, and cuticular softening of silkworm larvae^40^ and reduced flight activity and brood-rearing activity occurred in bees exposed to $S0_2$^41^, but little information is available on the modes of action and toxicology of sulfur compounds in insects. In addition to the major classes of pollution affecting insects, there are some references suggesting that arsenic^42^ have negative effects on insects and air pollutants are likely to impact olfactory-guided behavior^43^, thus reducing pollination services provided by insects^44^. Additionally, the presence of cadmium, copper, iron, nickel and other substances used in fertilizers were studied by Heliövaara, K. and Väisänen, R (1990) with pupae of different Noctuidae and Geometridae species, an Eriocraniidae population, about the cycle duration and newly hatched larval mortality rate from butterflies (Family Nymphalidae), which were feed on plants exposed to high carbon dioxide concentration^38^. Furthermore, there is a potential for air pollutants, particularly to negatively influence the searching behaviour of parasitoids^45^, but effects of air pollution on insect populations are poorly understood.

To date, considerable efforts have been made to the conservation of endangered butterfly species in certain cities and their surroundings^46,47^. Previous studies and initiatives have demonstrated that butterfly conservation in urban areas is a viable endeavor, given that many species exhibit the ability to thrive in these environments^48,49^. Envisioning a positive impact in urban environments for butterflies, creative urban planning and management strategies, such as habitat design and the cultivation of native host and nectar-rich plants, could enhance and improve urban habitats for butterflies. However, the success of these actions require careful monitoring and should be based in prior knowledge of the biology and ecology of the target species^50^. The reasons for using insect species, butterflies included, as indicator are: (1) The use of several different taxa of different habitats gives more robust results, (2) the quantitative indicator value needs to be associated with the bioindicators, (3) there is similarity between different landscape features, (4) there is comparison of community, (5) these taxa can be reliably identified, sampled, and quantified, and (6) more than one family surely indicate species richness of an order^51^. They exhibit a rapid and sensitive response to accumulation of heavy metals^52^. Moreover, due to their dynamic reproductive cycles, they respond rapidly to changes in the vegetation and climate, and they are conspicuous, therefore they can easily be observed and sampled throughout the year and have a well-known taxonomy^53^. They are also used as models in research on population ecology and behavior^54^.

Air-pollution has frequently been suggested as a cause of the decline of some butterfly species, but the suggestion are based mainly on lowered species richness close to industrial areas in Europe. Additionally, the effects of the urbanization on butterflies and other insects have scarcely been appraised in detail^51^ and there have been frequent calls, in vain, for research on the direct effect of air-pollution on Lepidoptera, with the recent investigations predominantly focusing on the indirect effects of air pollution (climate change)^55,56^. Furthermore, no experiments have been conducted to directly study the effects of air pollution on butterfly ecology, especially regarding the Sedimentable Particulate Matter (SPM). Since urbanization, which is associated with habitat degradation and increased air pollution^22^, and because of other factors, such as that the insects are popularly used as bioindicators^30^ including the group Lepidoptera^32^, secondly, because negative effects on butterflies are caused by urbanization^57^ that can lead to the reduction of some butterfly species^55^, furthermore, because butterflies are the most sensitive wildlife group to urbanization^58^ and due to what Sildanchandra and Crane (2000) said about the effects of air pollution on some insects, which are growth inhibition, developmental abnormalities, reduced reproduction, reduced survival and decreased hatchability, and that the direct effects of the urbanization on butterflies have scarcely been appraised in detail^55^, including just some studies about the effects of urbanization^51^, and other few the effects of air pollution on butterfly^56^, but no experiments have been conducted to investigate the direct effects of the Sedimentable Particulate Matter (SPM) on the group of Lepidoptera, this study, which is conducted with a species of Heliconiinae relatively common in urban green areas of the city of Vitória, ES (Brazil) called *Heliconius ethilla* (Godart, 1819), represents a pioneer experiment on the investigation of the direct effects of SPM on this model urban species of butterfly. Our objective was to investigate the effects of the sedimentable particulate matter on the survival of *H. ethilla*, and what were the direct effects. Our hypothesis is that the greater the concentration of SPM, the worse is the effect on the survival of *H. ethilla*.

## Results

### The treatment group had a higher probability of death and lower probability of survival to the next stage compared to the control group

**Fig. 1:**
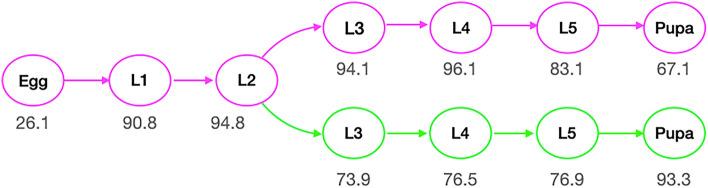
Graphs of the life cycle of the control and treatment groups from egg to pupa of *Heliconius ethilla*. with the percentage of the probability of survival to the next stage p(x).

Based on the observed results, the treatment group exhibited significantly lower survival probabilities, p (x), and and higher death probabilities, q(x), compared to the control group in the L3, L4, and L5 stages, as shown in Fig. 1 and Table 1, in Supplementary Materials.

### The treatment group exhibited significantly longer developmental times across all larval stages compared to the control group

**Fig. 2:**
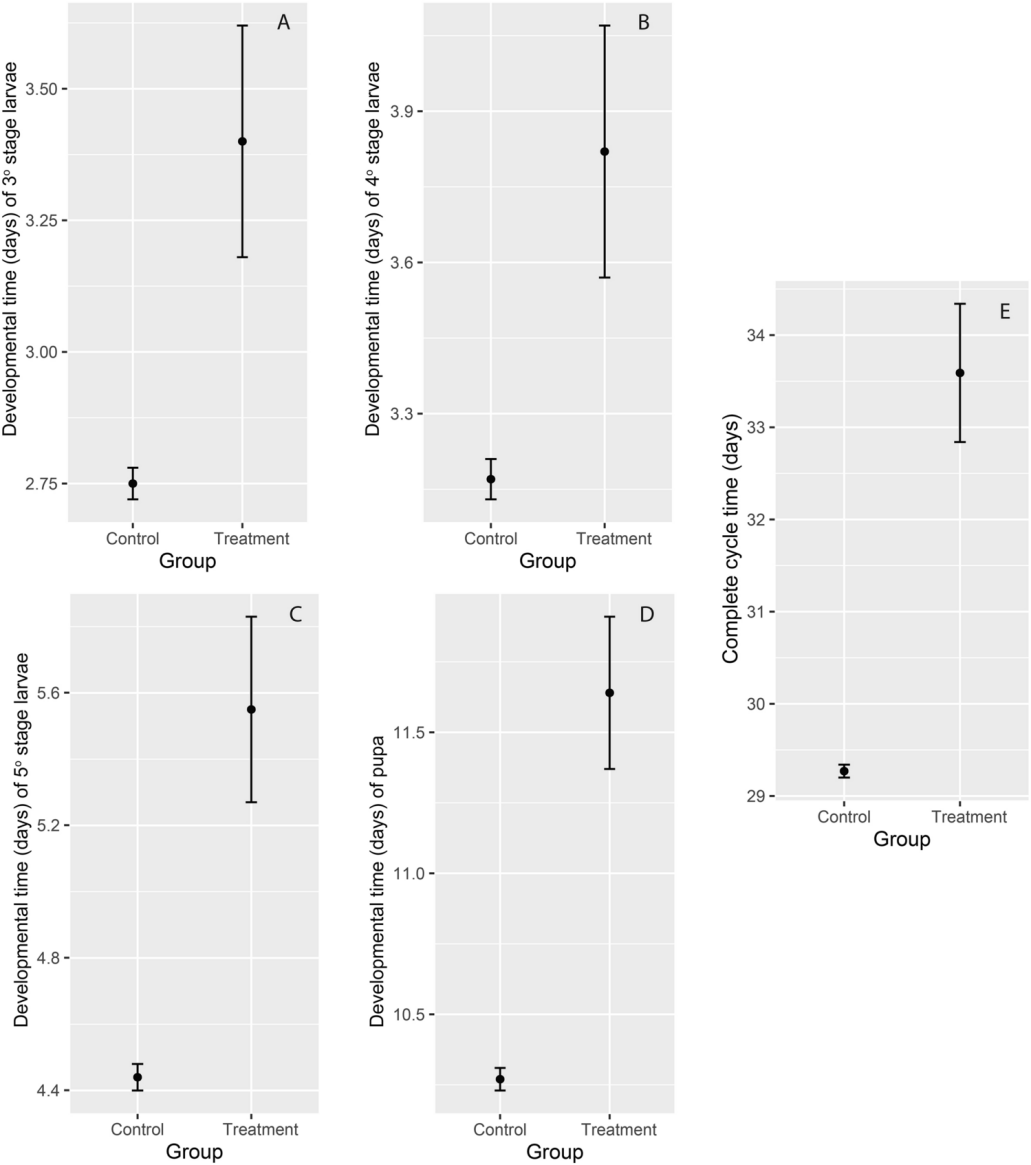
Graphs of the developmental time in days of the control and treatment groups of L3, L4, L5 and Pupa stages and the complete life cycle of *Heliconius ethilla*, in which, the L3, L4, L5 and Pupa stages are represented respectively in (**A**), (**B**), (**C**) and (**D**), and the complete life cycle is represented in (**E**). The dots represent the means and the error bars represent the 95% confidence intervals.

Developmental time was significantly longer in the treatment group compared to the control group, with the complete life cycle averaging 33.59 days for the treatment group versus 29.27 days for the control group, as shown in Fig. 2E and Table 2, in Supplementary Materials. The larvae passed through the stages faster in the control group compared to the treatment group, as we show in Fig. 2A–D, and in Table 2 in Supplementary Materials. The prolonged development in the treatment group reflects the impact of the experimental conditions on larval and pupal growth.

### The SPM significantly affected the size, weight, and survival of caterpillars in the treatment group

**Fig. 3:**
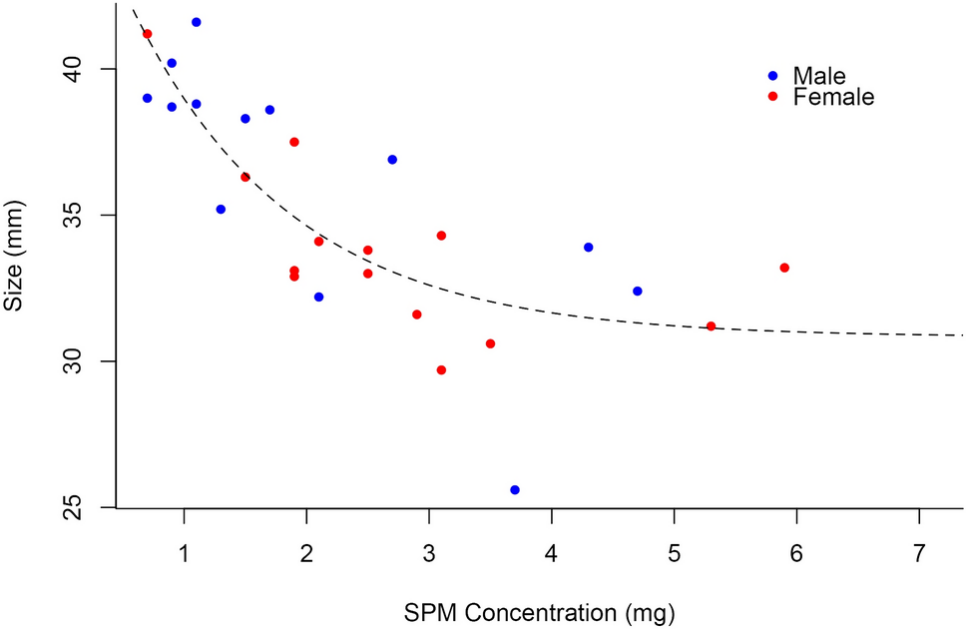
Graph of the adult size of *Heliconius ethilla* with the increasing concentration of SPM.

**Fig. 4:**
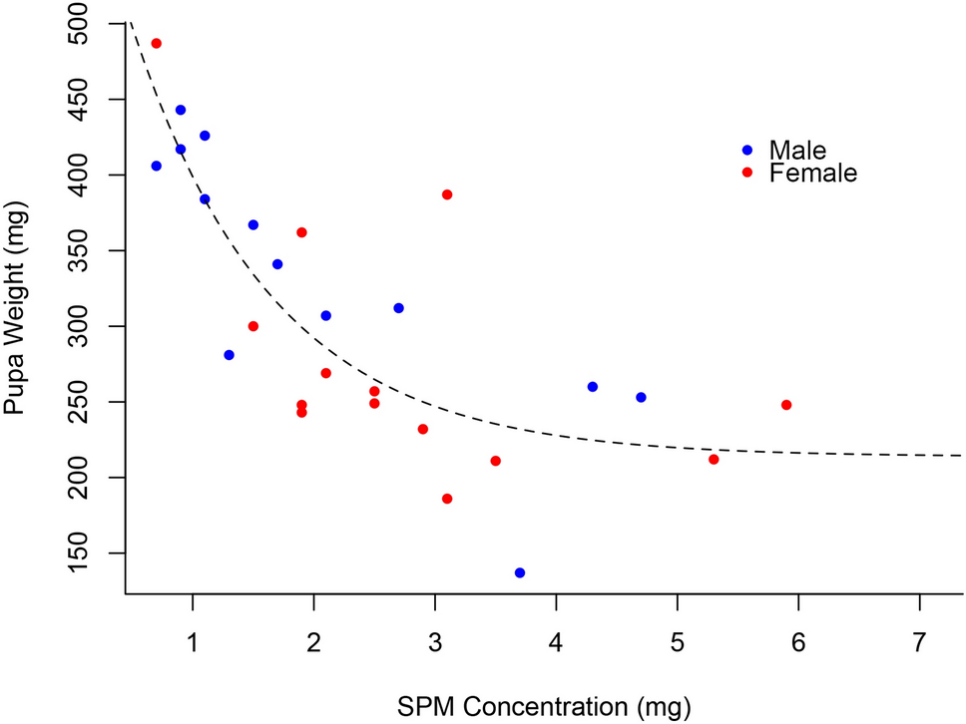
Graph of the weight of the pupa of *Heliconius ethilla* with the increasing concentration of SPM.

**Fig. 5:**
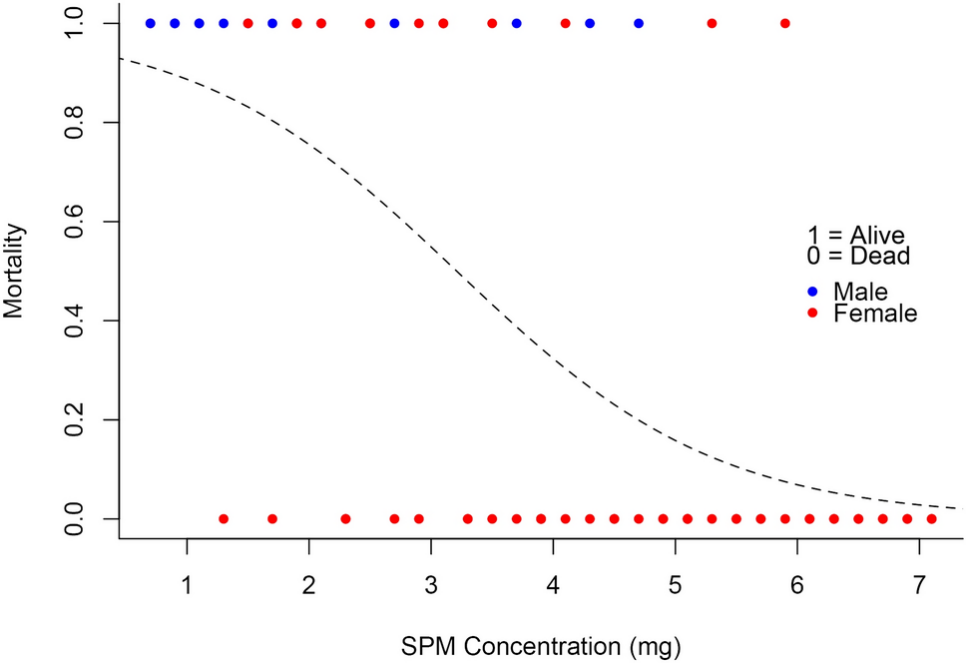
Graph of the Mortality of the individuals of *Heliconius ethilla* with the increasing concentration of SPM.

Our analysis revealed a strong negative correlation between adult size and SPM concentration, with size decreasing as SPM levels increased (Fig. 3, Table 1). Similarly, pupal weight showed a marked reduction with higher SPM concentrations (Fig. 4, Table 2). Mortality rates also increased with SPM levels, with higher concentrations resulting in significantly more deaths (Fig. 5, Table 3). These findings confirm the detrimental impact of SPM on the growth and survival of *H. ethilla*.

**Table 1:** Results of the negative exponential function of three parameters (a, b and c) of the size of the adults versus SPM concentration, with standard error (Std. error), t value and p-value.

Parameters	Estimate	Std. error	t value	p-value
a	30.8255	1.4674	21.007	$2*E^{-16}$
b	− 17.4639	3.9082	− 4.469	$1.6*E^{-4}$
c	30.8255	1.4674	21.007	0.02199

**Table 2:** Results of the negative exponential function of three parameters (a, b and c) of the pupa weight versus SPM concentration, with standard error (Std. error), t value and p-value.

Parameters	Estimate	Std. error	t value	p-value
a	213.7489	24.390	8.764	$2.22*E^{-9}$
b	− 436.505	97.090	− 44.960	$1.18*E^{-4}$
c	0.858	0.2824	3.039	0.00523

The Proportion Tests revealed no significant differences between the control and treatment groups in the proportion of individuals surviving to stages L3, L4, L5, or Pupa (Table 4). Similarly, the probability of surviving to the next stage, p(x), and the probability of death, q(x), showed no substantial differences across these stages between groups (Table 4). However, differences in life expectancy, e(x), were observed in L3 and L4 (p-values = 0.04316 and 0.03606, respectively), though no significant differences were found for L5 or Pupa (Table 4).

**Table 3:** Results of the logistic regression function of the survival of the caterpillars of *Heliconius ethilla* versus SPM concentration, with standard error (Std. error), z value and p-value.

Logistic regression	Estimate	Std. error	z value	p-value
Intercept	2.2952	0.8076	3.709	$2.08*E^{-4}$
Concentration	− 0.933	0.2178	− 4.286	$1.82*E^{-5}$

**Table 4:** Results of the Proportion Test of the l(x), p(x), q(x) and e(x) of the stages L3, L4, L5 and Pupa with X-Square Value and p-value.

Parameter	Stage	X-Square Value	p-value
l(x)	L3	0.35261	0.5526
	L4	0.0026322	0.9591
	L5	$3.4706*E^{-30}$	1
	Pupa	$4.5933*E^{-31}$	1
p(x)	L3	0.067353	0.7952
	L4	0.19303	0.6604
	L5	0.38624	0.5343
	Pupa	1.5617	0.2114
q(x)	L3	$2.3293*E^{-30}$	1
	L4	$3.9958*E^{-30}$	1
	L5	$3.5037*E^{-30}$	1
	Pupa	$2.1178*E^{-31}$	1
e(x)	L3	4.0891	0.04316
	L4	4.3941	0.03606
	L5	3.2239	0.07257
	Pupa	$2.3482*E^{-31}$	1

Although we conducted proportion tests and initially observed differences in life expectancy e(x), of the L3 and L4 stages (as shown in Table 4), it is important to note that due to the multiple comparisons we made, the Bonferroni Correction, a more accurate way to determine the statistical significance of our previous proportion tests comparisons, showed that there were no differences between the control and treatment groups (new p-value = 0.003125). Despite the absence of detectable variations in the life table parameters, as there were no substantial differences in the p(x), q(x), l(x) and e(x), noteworthy distinctions did emerge in terms of developmental time, mortality, pupal weight and body size.

## Discussion

Various mortality factors can interact, potentially resulting in additive or antagonistic changes in mortality^59^. Most larval mortality occurs during the first instar and the causes of this mortality are often unclear. Neonates must promptly establish feeding sites shortly after hatching^60–62^, and have to deal with a variety of challenges, including leaf hairs and trichomes^63^, surface waxes^64^, hard plant parts^65^, laticifers, glands or tissues filled with allelochemicals^66–68^, as well as potential threats from predators^69^ and leaf micro-flora, which is comprised by bacteria, fungi and other microbes^70,71^. The larvae, especially those reared in captivity, are more susceptible to infections from viruses and bacteria^72^. Additionally, plant pathogens may also alter host quality^73^. Extreme weather conditions, such as storms, have also been observed by Gilbert, L.E. (1975) as a contributing factor to population regulation in Heliconius species.

The food stress induced by increasing SPM concentrations likely reduced growth rates and prolonged developmental periods. According to Bauerfeind and Fischer (2005) the food stress during larval stage significantly reduces the Lepidoptera growth rate, pupal and adult mass, and prolongs the larval development time in Lepidoptera. They suggested that Lepidoptera larvae facing food stress compensate for temporarily reduced nutrient intake by extending the larval period. That result is believed to be universal and generally predicted by life-history models^74–77^. Tan, Y.Q and collaborators (2018) also observed in their experiment using haze smoke, that larvae exposed to smoke took longer time to reach the pupal stage compared to the larvae in the control group. Moreover, the authors also observed that the smoke reduced the probability of larvae surviving to the pupal stage and, consequently, to butterfly emergence^78^. In addition, Bauerfeind and Fischer (2005) also observed that larval food limitation not only had negative effects on the growth rate and longer developmental duration but it also led to a reduction in the fecundity and reproductive rates, mediated through a reduction in body size.

Although we do not use fecundity or reproductive rate, based on the observation of these previously mentioned authors and comparing with what we observed in our experiment with *H. ethilla*, we can infer a reduction in the fecundity and natality rate due to the reduction in the body size, probably because the smaller the body, the smaller the space to produce eggs and also due to what Sildanchandra and Crane (2000) said about the increasing appearance of developmental abnormalities and decreased hatchability. Tan, Y.Q and collaborators (2018) also observed that the pupae from individuals reared on smoked-plants were also smaller than the ones reared on plants not exposed to the haze, indicating that the smoke affected pupal weight. SPM similarly reduced larval survival and pupal emergence and also caused the developmental abnormalities, aligning with previous findings. Based on these observations, we can expect consequently, negative effects on the long-term species survival, particularly in a scenario in which air pollution concentrations is increasing over time, including SPM and other components of air pollution.

The reduction in pupal weight with increasing SPM concentration suggests that adults will also have a reduced size, with potential implications for fecundity. Comparing these effects observed by Bauerfeind and Fischer (2005) and based on our observations on the survival rate and reduction in the pupal weight and speculations about the fecundity and natality rates it could be expected that *H. ethilla* would experience similar effects observed by Bauerfeind and Fischer (2005) and Sildanchandra and Crane (2000). Consequently, our results and speculations allied with the observations of these authors previously mentioned, may indicate a reduction in the population size of the species and consequent decrease in the chances of long-term survival due to the increase in mortality at different stages of life and probable reduction in the reproduction and natality rates. Mulder and collaborators (2005) demonstrated that the butterflies and their host plant species have low tolerance to pollution, highlighting a correlation between the sensitivity of the butterflies and their hosts. These same authors emphasised the potential coexistence of indirect effects of the pollutants on the adult butterflies and a direct effect of xenobiotics on the larvae. That’s what *H. ethilla* could be facing.

Based on the results and the discussion, it is evident that sedimentable particulate matter (SPM) substantialy and negatively influences the population dynamics and life history traits of *H. ethilla*, a butterfly species found in the southeastern region of Brazil. Our study observed a reduction in the survival rates, longer time of development, and decreased pupal weight as SPM concentration increased. These findings are consistent with previous research on other Lepidoptera species facing food stress and exposure to pollutants. The lower survival rates and extended developmental periods observed in the treatment group suggest that the presence of SPM in the environment, creates food stress for *H. ethilla* larvae. As observed in similar studies, the larvae use compensatory mechanisms, extending their development period to counteract reduced nutrient intake caused by pollution. This response holds important implications for the overall fitness and reproductive success of the butterfly population. It is highly possible that the observed reduction in pupal weight will mean the individuals will have smaller adult sizes, which, in turn, could lead to reduced fecundity and natality rates.

Beyond the lethal effects, observed mostly and more abruptly in higher concentrations of SPM, even in lower concentration mortality occurred. Sublethal effects that arise in lower concentrations of SPM can be many times underestimated, but they still cause long-term mortality. The consequences of these effects could be severe on the long-term, potentially leading to a decline in the population size of *H. ethilla*. In a scenario where air pollution continues to increase over time, the observed mortality at different life stages, coupled with reduced reproductive potential, could concatenate and intensify, ultimately threatening the long-term survival of this butterfly species. This aligns with similar studies, highlighting the low tolerance of butterflies, their host plants, and their sensitivity to pollutants. The coexistence of indirect effects on adults and direct effects on larvae due to xenobiotics underscores the complex challenges *H. ethilla* faces in its environment.

The investigation we did is extremely vital given the lack of information on the decline of insect in South America. Most data on the decline of insects comes from the Northern Hemisphere^79^, mostly from countries from Europe and North America, since those regions have the most comprehensive historical records that allow comparisons of biodiversity on a temporal scale^80^, while there is a noteworthy taxonomic and geographic^79^ data availability gaps for the Southern Hemisphere^81^, which includes South America, since it has the most incomplete inventory for insects^79^, even though the region hosts essential habitats that are considered global biodiversity hotspots^82^.

In conclusion, our findings highlight the significant negative impact of sedimentable particulate matter on the survival, development, and reproductive potential of *H. ethilla*. As air pollution continues to be a growing concern in urban and natural environments, these findings emphasize the importance of addressing pollution mitigation strategies to protect not only *H. ethilla* but also other sensitive species that rely on similar habitats not only in the southeastern region of Brazil, but also all around the World.

## Methods

### Experimental setup

The bionomic data of *Heliconius ethilla* were collected at the Entomological and butterfly house within the Laboratory of the Environmental Education Center of a Siderurgical Industry, located in the municipality of Serra, Espírito Santo, Brazil. To determine the viability and the time of development of the eggs until the adult emergence, eggs were collected and kept in air-conditioned chambers (B.O.D.) at 25$\pm$0,3$^{\circ }$C with a photoperiod of 12 hours until adult emergence, if there was any adult emergence. We started our experiment with 3255 eggs, from which 69 stage 3 caterpillars were randomly separated from the control group as the treatment group and the 732 remaining stage 3 caterpillars were maintained as the control group.

In this study, we employed the American Society for Testing and Materials (ASTM) method, specifically the Standard Test Method for Collection and Measurement of Dustfall (Settleable Particulate Matter) denoted as ASTM D1739 - 98 (reapproved on 2004), for the collection of settleable particulate matter used in our investigation. We monitored for six months the deposition of Sedimentable Particulate Matter (SPM) and applied standard gravimetric analysis to determine the mean daily rate deposition of SPM in the environment applying the following formula:

$$\begin{aligned} Q=(3*m)/t*\pi *(D/2)^2 \end{aligned}$$

Where:

**Q** is the sedimentable dust rate in milligrams per square centimeter for a 30-day collection period.

**m** is the mass of collected dust in milligrams.

**t** is the period of collection in days.

**D** is the assumed diameter of the collection flask, set to 100 millimeters.

This formula allows the quantification of the sedimentable dust rate per unit area, taking into account the mass of collected dust, period in which happened the dust collection, and the assumed diameter of the collection flask. The use of milligrams ensures a more precise resolution of the mass measurement, and the result is presented in milligrams per square centimeter to standardize the rate over the assumed collection area.

**Fig. 6:**
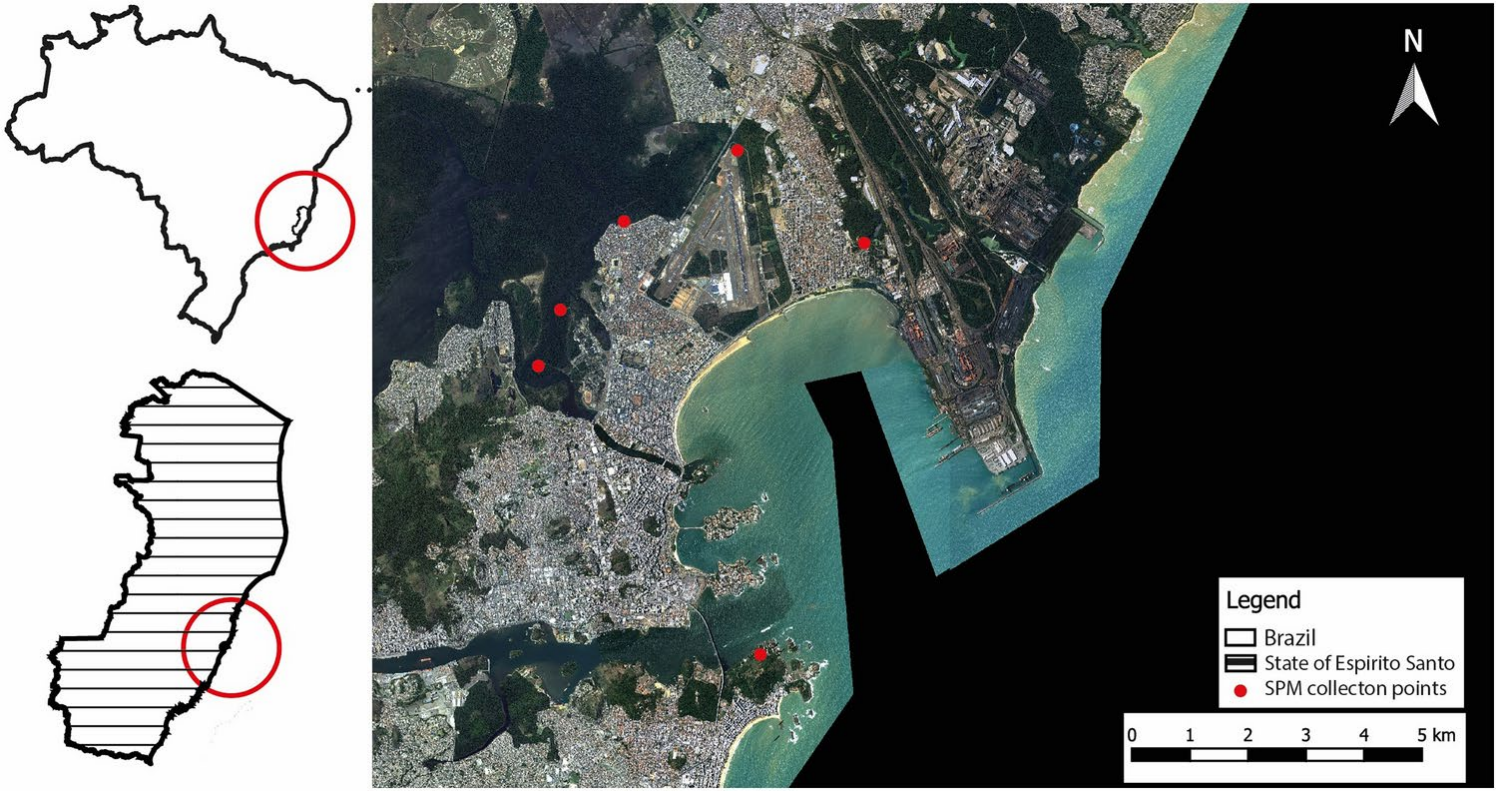
Map of Greater Vitória with the location of the six collection points (red dots) of sedimentable particulate matter. This map was created using Qgis 3.36.3.

The six sedimentable particulate matter collection points were independently installed by us near the locations of the automatic atmospheric air quality monitoring network in the Greater Vitória region, managed by the Instituto Estadual de Meio Ambiente e Recursos Hídricos (IEMA). The specific location of the points and the map of the region can be seen in Fig. 6. No physicochemical analysis were carried out because the composition of sedimentable particulate matter (SPM) in the atmospheric air surrounding steel industries is a complex and presumed merging of solid and liquid particles that settle when air speed decreases. While the specific composition of SPM can vary based on geographical location, industrial practices, and specific processes within the steel production, common components include metallic particles such as iron, aluminum, zinc, and lead, derived from metallurgical processes. Additionally, particles originated from the combustion of coal or coke, including carbon, ashes, and related byproducts, contribute to the SPM formation. The emissions from steel manufacturing processes, such as smelting and rolling, generate particulate matter usually composed by a mixture of metal oxidated residues and dust.

**Fig. 7:**
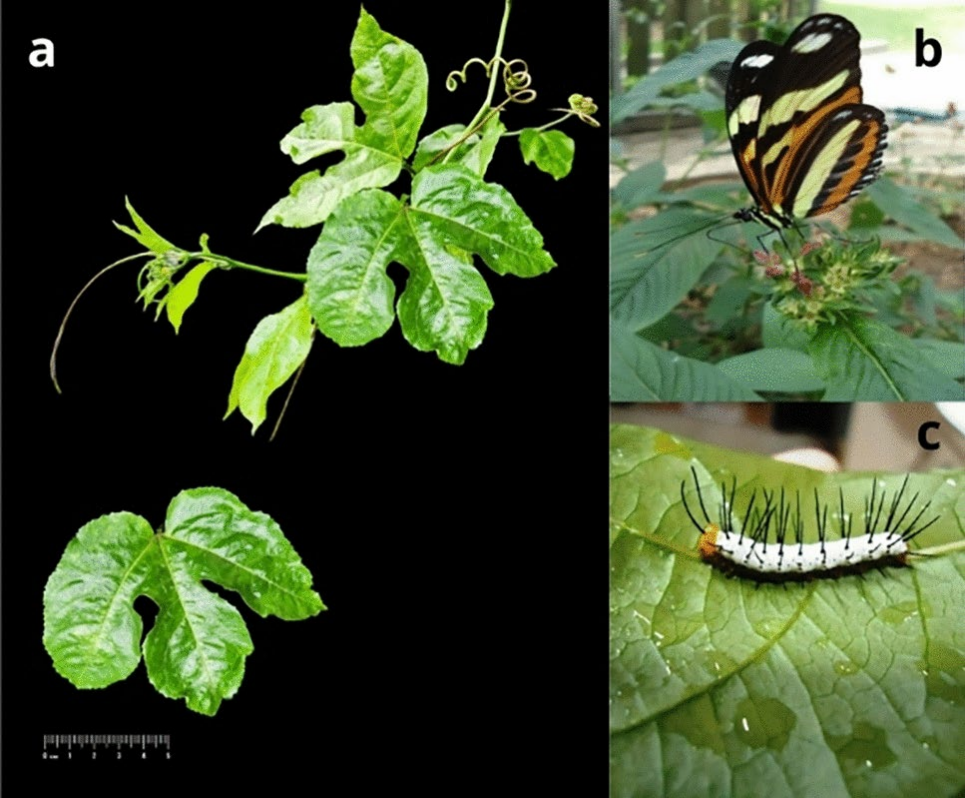
Photos of the species of the plant and Lepidoptera used in the experiment. (**a**) Photo of *Passiflora edulis*, (**b**) adult of *Heliconius ethilla* and (**c**) L5 stage caterpillar of *Heliconius ethilla*.

The caterpillars were fed with *Passiflora edulis* Sims f. flavicarpa Deg. leaves (Fig. 7a), with SPM added to the experimental group’s leaves before being offered to the caterpillars in an increasing concentration according to the age of the caterpillar and the leaf, simulating the gradual deposition of this material in the environment.

### Building the life tables

The life tables and graphs of the life cycle were elaborated with the data of mortality and survival of the individuals of the control and experimental groups following the methodologies of Rockwood (2006), Bellows Jr and collaborators (1992) and Gotelli (2009), where x indicates the stages of the life cycle (egg, five larval stages designated from L1 to L5, pupa and adult); S(x) is the number of individuals at the beginning of each stage; l(x) indicates the proportion of the population surviving to stage x; p(x) is the probability of surviving stage and q(x) is the probability of death. Additionally, the proportion of the population that lived until stage x which is L(x), the proportion of the population that lived until stage x and in all subsequent phases which is T(x) and life expectancy e(x) were also calculated. Based on the mortality and survival profiles of the life table data, the life cycle charts were generated. Bellow are the formulas that were used to build the life tables:

Proportion of the population surviving to the stage (x):

$$\begin{aligned} l(x) =N_{x} /N_0 \end{aligned}$$

Probability of surviving stage:

$$\begin{aligned} p(x)=N_{x+1}/N_x \end{aligned}$$

Probability of death:

$$\begin{aligned} q(x)=1-p(x) \end{aligned}$$

Proportion of the population that lived until stage (x):

$$\begin{aligned} L(x) =l_x + l_{x +1}/2 \end{aligned}$$

Proportion of the population that lived until stage (x) and in all subsequent stages:

$$\begin{aligned} T(x)=L_x + L_{x+1} + L_{x+2}.... L_{x+n} \end{aligned}$$

Life expectancy:

$$\begin{aligned} e(x)=T_x /l_x \end{aligned}$$

Although the Lepidoptera has 8 stages in total, we focused our investigation on L3 to L5 stages and Pupa stage because various factors contribute to Lepidoptera mortality^59^, including in Heliconius populations^83^. To avoid unwanted and confounding death factors observed by many authors^66–72,83^, since many sources are responsible for the mortality and because the first meal taken by a larva is normally its own chitinous egg shell^72^, we started the treatment group with the third instar stage (L3). Additionally, this choice stems not only because of the numerous mortality factors observed in their early stages, but also because starting from the third instar, the caterpillars become slightly larger and more robust, making easy to handle them and reducing the risk of injury on the larvae during manipulation. Furthermore, the chosen host plant belongs to the Passifloraceae family and it exhibits a climbing growth habit, with its vegetative tips and initial leaves adopting contorted shapes to adapt to the surrounding environment. Since new leaves emerge every three days, there is a correlation between the optimal size of the caterpillar and the onset of a more horizontally positioned open leaf, presumed to mark the beginning the sedimentable particulate matter deposition. The photos of the *H. ethilla* adult and L5 stage caterpillar can be seen respectively in the Fig. 7b,c.

### Statistical tests

We used the R platform v. 4.2.3^84^ to (1) build the life tables and to adjust non-linear equation (negative exponential function of three parameters) for the size of adults and the pupa weight with increasing concentration of SPM and to (2) adjust the logistic regression to evaluate the survival of the caterpillars with the increasing concentration of SPM. In our study, the selection of a three-parameter negative exponential nonlinear regression model was driven by the nature of our data and the underlying biological processes being investigated. The choice of this model was based on the observation that the relationship between the variables under consideration did not conform to a simple linear or polynomial pattern. Instead, our data exhibited a declining trend with a rapid initial decrease followed by a gradual leveling off. The three-parameter negative exponential model allowed us to capture the non-linear decay pattern observed in our dataset. The three parameters (a, b and c) provided flexibility in fitting the model to the intricacies of the biological system under study. The parameter **a** determined the initial amplitude of the response, **b** controlled the rate of decay, and **c** accounted for any offset or residual baseline. We used Qgis 3.36.3 “Maidenhead”^85^ to build the study area map showing the sampling points of the SPM.

Additionally, we also generated graphical representations with the ggplot2 package v. 3.4.2^86^ of the developmental time of the control and treatment groups of the L3, L4, L5 and pupa stages and complete life cycles. From the results of the life table we also used R platform v. 4.2.3^84^ to do proportion tests to compare if there were differences in l(x), p(x), q(x) and e(x). Since we did multiple comparison tests with the same data we also used the Bonferroni Correction (new p-value = 0.003125) to be able to determine more accurately the significance of the statistical tests applied. We also calculated the 95% Confidence Interval of the time of development with which we used as a statistical method for comparison between the control and treatment groups.

## Supplementary Information

Supplementary Tables.

## Data Availability

The original data is available and stored in the Google Drive in Google Sheet format provided in the following link http://tinyurl.com/3h752tum

## References

[1] Qadir, A. & Malik, R. N. Assessment of an index of biological integrity (IBI) to quantify the quality of two tributaries of river Chenab, Sialkot, Pakistan. *Hydrobiologia*10.1007/s10750-008-9637-0 (2009).

[2] Atafar, Z. et al. Effect of fertilizer application on soil heavy metal concentration. *Environ. Monit. Assess.*10.1007/s10661-008-0659-x (2010). 19058018 10.1007/s10661-008-0659-x

[3] Lee, C. S. L., Li, X., Shi, W., Cheung, S. C. N. & Thornton, I. Metal contamination in urban, suburban, and country park soils of Hong Kong: A study based on GIS and multivariate statistics. *Sci. Total Environ.*10.1016/j.scitotenv.2005.03.024 (2006). 15913711 10.1016/j.scitotenv.2005.03.024

[4] EPA, U. National ambient air quality standards (NAAGS). Tech. Rep., U.S Environmental Protection Agency (2014). https://www.epa.gov/sites/default/files/2015-02/documents/criteria.pdf (Accessed 15 Dec 2023).

[5] EEA. Air quality in Europe–2020 report, EEA report no 09/2020. Tech. Rep., European Environment Agency (2020). https://www.eea.europa.eu/publications/air-quality-in-europe-2020-report (Accessed 15 Dec 2023).

[6] WHO. Air quality guidelines: Global update 2005. report on a working group meeting, bonn, germany, 18-20 october 2005. Tech. Rep., World Health Organization. Regional Office for Europe (2005). https://iris.who.int/handle/10665/349878 (Accessed 02 Dec 2023).

[7] Holgate, S., Koren, H., Samet, J. & Maynard, R. *Air Pollution and Health* 1st edn. (Academic Press, 1999).

[8] Larssen, L., S. & Hagen. Air quality in Europe, 1993 a pilot report. Tech. Rep., European Environment Agency (1996). https://www.eea.europa.eu/publications/2-9167-057-X (Accessed 08 Dec 2023).

[9] WHO. Air quality guidelines: Global update 2005: particulate matter, ozone, nitrogen dioxide and sulfur dioxide. Tech. Rep., World Health Organization. Regional Office for Europe (2006). https://www.who.int/publications/i/item/WHO-SDE-PHE-OEH-06.02 (Accessed 07 Dec 2023).

[10] Organization, W. H. Who global air quality guidelines: particulate matter (pm2.5 and pm10), ozone, nitrogen dioxide, sulfur dioxide and carbon monoxide. Tech. Rep., World Health Organization (WHO) (2021). https://www.who.int/publications/i/item/9789240034228 (Accessed 15 Dec 2023). 34662007

[11] Schraufnagel, D. The health effects of ultrafine particles. *Exp. Mol. Med.* **52**, 311–317. 10.1038/s12276-020-0403-3 (2020). 32203102 10.1038/s12276-020-0403-3 PMC7156741

[12] Chen, R. et al. Beyond : The role of ultrafine particles on adverse health effects of air pollution. *Biochim. et Biophys. Acta (BAA) Gen. Subj.* **2844–2855**, 2016. 10.1016/j.bbagen.2016.03.019 (1860). 10.1016/j.bbagen.2016.03.019 26993200

[13] Panel, H. R. *Understanding the Health Effects of Ambient Ultrafine Particles* (Health Effect Institute, 2013).

[14] Terzano, C., Di Stefano, F., Conti, V., Graziani, E. & Petroianni, A. Air pollution ultrafine particles: Toxicity beyond the lung. *Eur. Rev. Med. Pharmacol. Sci.* **14**, 809–821 (2010). 21222367

[15] Xue, J. et al. Seasonal and annual source appointment of carbonaceous ultrafine particulate matter (pm 0.1) in polluted California cities. *Environ. Sci. Technol.* **53**, 39–49. 10.1021/acs.est.8b04404 (2019). 30452867 10.1021/acs.est.8b04404

[16] Rahman, M. M., Mazaheri, M., Clifford, S. & Morawska, L. Estimate of main local sources to ambient ultrafine particle number concentrations in an urban area. *Atmos. Res.*10.1016/j.atmosres.2017.04.036 (2017).

[17] Ge, Y. et al. High spatial resolution land-use regression model for urban ultrafine particle exposure assessment in Shanghai, China. *Sci. Total Environ.*10.1016/j.scitotenv.2021.151633 (2022). 34785221 10.1016/j.scitotenv.2021.151633

[18] Heinzerling, A., Hsu, J. & Yip, F. Respiratory health effects of ultrafine particles in children: A literature review. *Water Air Soil Pollut.*10.1007/s11270-015-2726-6 (2016). 26783373 10.1007/s11270-015-2726-6 PMC4714792

[19] Romieu, I. *et al.* Multicity study of air pollution and mortality in Latin America (the Escala study). *Research report (Health Effects Institute)* (2012). 23311234

[20] WHO. Burden of disease from ambient air pollution for 2016 (version 2 updated in april 2018). Tech. Rep., World Health Organization (WHO) (2018). https://cdn.who.int/media/docs/default-source/air-pollution-documents/air-quality-and-health/aap_bod_results_may2018_final.pdf (Accessed 06 Dec 2023).

[21] Collaborators, G. R. F. Global burden of 87 risk factors in 204 countries and territories, 1990–2019: a systematic analysis for the global burden of disease study 2019. *Lancet*10.1016/S0140-6736(20)30752-2 (2020). 10.1016/S0140-6736(20)30752-2 PMC7566194 33069327

[22] McKinney, M. L. Urbanization, biodiversity, and conservation. *BioScience*10.1641/0006-3568(2002)052[0883:UBAC]2.0.CO;2 (2002).

[23] Germaine, S. S. & Wakeling, B. F. Lizard species distributions and habitat occupation along an urban gradient in Tucson, Arizona, USA. *Biol. Conserv.*10.1016/S0006-3207(00)00115-4 (2001).

[24] Clark, P. J., Reed, J. M., Tavernia, B. G., Windmiller, B. S. & Regosin, J. V. Urbanization effects on spotted salamander and wood frog presence and abundance. In *Urban Herpetology*, 1–9 (Society for the Study of Amphibians and Reptiles, 2008).

[25] Riley, S. P. et al. Effects of urbanization and habitat fragmentation on bobcats and coyotes in southern California. *Conserv. Biol.*10.1046/j.1523-1739.2003.01458.x (2003).

[26] Miller, J. R., Wiens, J. A., Hobbs, N. T. & Theobald, D. M. Effects of human settlement on bird communities in lowland riparian areas of Colorado (USA). *Ecol. Appl.*10.1890/1051-0761(2003)13[1041:EOHSOB]2.0.CO;2 (2003).

[27] Lee, P. F., Ding, T. S., Hsu, F. H. & Geng, S. Breeding bird species richness in Taiwan: Distribution on gradients of elevation, primary productivity and urbanization. *J. Biogeogr.*10.1046/j.0305-0270.2003.00988.x (2004).

[28] Gibb, H. & Hochuli, D. F. Habitat fragmentation in an urban environment: Large and small fragments support different arthropod assemblages. *Biol. Conser.*10.1016/S0006-3207(01)00232-4 (2002).

[29] Tscharntke, T., Steffan-Dewenter, I., Kruess, A. & Thies, C. Characteristics of insect populations on habitat fragments: A mini review. *Ecol. Res.*10.1046/j.1440-1703.2002.00482.x (2002).

[30] Davis, A. J. et al. Dung beetles as indicators of change in the forests of northern Borneo. *J. Appl. Ecol.* **38**, 5. 10.1046/j.1365-2664.2001.00619.x (2001).

[31] Azam, I. et al. Evaluating insects as bioindicators of heavy metal contamination and accumulation near industrial area of Gujrat, Pakistan. *BioMed Res. Int.*10.1155/2015/942751 (2015). 26167507 10.1155/2015/942751 PMC4488521

[32] Kumar, A. Butterfly abundance and species diversity in some urban habitats. *Int. J. Adv. Res.* **2**, 367–374 (2014).

[33] Soga, M. & Koike, S. Relative importance of quantity, quality and isolation of patches for butterfly diversity in fragmented urban forests. *Ecol. Res.*10.1007/s11284-011-0896-2 (2012).

[34] Soga, M. & Koike, S. Mapping the potential extinction debt of butterflies in a modern city: Implications for conservation priorities in urban landscapes. *Anim. Conserv.*10.1111/j.1469-1795.2012.00572.x (2013).

[35] Bergerot, B., Fontaine, B., Julliard, R. & Baguette, M. Landscape variables impact the structure and composition of butterfly assemblages along an urbanization gradient. *Landsc. Ecol.*10.1007/s10980-010-9537-3 (2011).

[36] Bergerot, B., Merckx, T., Van Dyck, H. & Baguette, M. Habitat fragmentation impacts mobility in a common and widespread woodland butterfly: do sexes respond differently?. *BMC Ecol.*10.1186/1472-6785-12-5 (2012). 22540674 10.1186/1472-6785-12-5 PMC3430564

[37] Soga, M. & Koike, S. Patch isolation only matters for specialist butterflies but patch area affects both specialist and generalist species. *J. For. Res.*10.1007/s10310-012-0349-y (2013).

[38] Mauricio da Rocha, J. R., De Almeida, J. R., Lins, G. A. & Durval, A. Insects as indicators of environmental changing and pollution: A review of appropriate species and their monitoring. *Holos Environ.*10.14295/holos.v10i2.2996 (2010).

[39] Sildanchandra, W. & Crane, M. Influence of sexual dimorphism in chironomus riparius meigen on toxic effects of cadmium. *Environ. Toxicol. Chem.* **19**, 2309 (2000).

[40] Kuribayashi, S. Environmental pollution of sericulture and its countermeasures. *Sanshi Kagaku To Gijutsu* **10**, 48–49 (1971).

[41] Hillman, R. C. *Biological effects of air pollution on insects, emphasizing the reactions of the honey bee (Apis melilifera L.) to sulfur dioxide*. Ph.D. thesis, Penn. State Univ (1972).

[42] Mueller, B. W. M. Damage to bees caused by arsenic- and fluorine-containing industrial flue gas. *Veterinaermed* **25**, 554–556 (1970). 5481027

[43] Dethier, V. G. *Chemical Insect Attractants and Repellents* (Hassell Street Press, 2021).

[44] Ryalls, J. M. et al. Anthropogenic air pollutants reduce insect-mediated pollination services. *Environ. Pollut.*10.1016/j.envpol.2022.118847 (2022). 35063287 10.1016/j.envpol.2022.118847

[45] Gate, I. M., McNeill, S. & Ashmore, M. R. Effects of air pollution on the searching behaviour of an insect parasitoid. *Water Air Soil Pollut.*10.1007/BF00477181 (1995).

[46] Murphy, D. D. & Weiss, S. B. Ecological studies and the conservation of the bay checkerspot butterfly, *Euphydrias editha bayensis*. *Biol. Conserv.* **46**, 183–200 (1988).

[47] Daniels, J. C. Cooperative conservation efforts to help recover an endangered South Florida butterfly. *Insect Conserv. Divers.*10.1111/j.1752-4598.2008.00039.x (2009).

[48] Snep, R. P. et al. How peri-urban areas can strengthen animal populations within cities: A modeling approach. *Biol. Conserv.*10.1016/j.biocon.2005.06.034 (2006).

[49] Kadlec, T., Benes, J., Jarosik, V. & Konvicka, M. Revisiting urban refuges: Changes of butterfly and burnet fauna in Prague reserves over three decades. *Landsc. Urban Plan.*10.1016/j.landurbplan.2007.07.007 (2008).

[50] Kremen, C., Merenlender, A. M. & Murphy, D. D. Ecological monitoring: A vital need for integrated conservation and development programs in the tropics. *Conserv. Biol.*10.1046/j.1523-1739.1994.08020388.x (1994).

[51] New, T. R. & Sands, D. P. Conservation concerns for butterflies in urban areas of Australia. *J. Insect Conserv.*10.1023/A:1024425515889 (2002).

[52] Cervera, A., Maymó, A. C., Sendra, M., Martínez-Pardo, R. & Garcerá, M. D. Cadmium effects on development and reproduction of *Oncopeltus fasciatus* (heteroptera: Lygaeidae). *J. Insect Physiol.*10.1016/j.jinsphys.2004.06.001 (2004). 15288207 10.1016/j.jinsphys.2004.06.001

[53] Brown, K. S. Conservation of neotropical environments: Insects as indicators. In *The Conservation of Insects and their Habitats*10.1016/b978-0-12-181370-3.50020-8 (Academic Press, 1991).

[54] Dessuy, M. B. & Morais, A. B. B. D. Diversidade de borboletas (lepidoptera, papilionoidea e hesperioidea) em fragmentos de floresta estacional decidual em santa maria, rio grande do sul, brasil. *Revista Brasileira de Zoologia*10.1590/s0101-81752007000100014 (2007).

[55] Corke, D. Are honeydew/sap-feeding butterflies (lepidoptera: Rhopalocera) affected by particulate air-pollution?. *J. Insect Conserv.*10.1023/A:1009670404398 (1999).

[56] Dennis, R. L. H. *Butterflies and Climate Change* (Manchester University Press, 1993).

[57] Ramírez-Restrepo, L. & MacGregor-Fors, I. Butterflies in the city: a review of urban diurnal lepidoptera. *Urban Ecosyst.*10.1007/s11252-016-0579-4 (2017).

[58] MacGregor-Fors, I. et al. Multi-taxonomic diversity patterns in a neotropical green city: a rapid biological assessment. *Urban Ecosyst.*10.1007/s11252-014-0410-z (2015).

[59] Johnson, M. T. & Gould, F. Interaction of genetically engineered host plant resistance and natural enemies of heliothis virescens (lepidoptera: Noctuidae) in tobacco. *Environ. Entomol.* **21**, 586–597. 10.1093/ee/21.3.586 (1992).

[60] Bernays, E. A., Chapman, R. F. & Woodhead, S. Behaviour of newly hatched larvae of chilo partellus (swinhoe) (lepidoptera: Pyralidae) associated with their establishment in the host-plant, sorghum. *Bull. Entomol. Res.*10.1017/S000748530001381X (1983).

[61] Cockfield, S. D. & Mahr, D. L. Consequences of feeding site selection on growth and survival of young blackheaded fireworm (lepidoptera: Tortricidae). *Environ. Entomol.*10.1093/ee/22.3.607 (1993).

[62] Hochberg, M. E. The within-plant distribution and feeding behaviour of heliothis armigera hübner (lep., noctuidae) on greenhouse tomatoes. *J. Appl. Entomol.*10.1111/j.1439-0418.1987.tb00523.x (1987).

[63] Haddad, N. M. & Hicks, W. M. Host pubescence and the behavior and performance of the butterfly papilio troilus (lepidoptera: Papilionidae). *Environ. Entomol.*10.1093/ee/29.2.299 (2000).

[64] Eigenbrode, S. D. & Shelton, A. M. Behavior of neonate diamondback moth larvae (lepidoptera: Plutellidae) on glossy-leafed resistant *Brassica oleracea* L. *Environ. Entomol.*10.1093/ee/19.5.1566 (1990).

[65] Lucas, P. W., Turner, I. M., Dominy, N. J. & Yamashita, N. *Mechanical Defences to Herbivory*10.1006/anbo.2000.1261 (2000).

[66] Compton, S. G. *Aganais speciosa* and *Danaus chrysippus* (lepidoptera) sabotage the latex defences of their host plants. *Ecol. Entomol.*10.1111/j.1365-2311.1987.tb00990.x (1987).

[67] Dussourd, D. E. & Eisner, T. Vein-cutting behavior: Insect counterploy to the latex defense of plants. *Science*10.1126/science.3616620 (1987). 3616620 10.1126/science.3616620

[68] Farrell, B. D., Dussourd, D. E. & Mitter, C. Escalation of plant defense: do latex and resin canals spur plant diversification?. *Am. Nat.*10.1086/285258 (1991).

[69] Heinrich, B. *Caterpillars. Ecological and Evolutionary Constraints on Foraging*, chap. How avian predators constrain caterpillar foraging, 224–247 (Chapman and Hall, 1993).

[70] Barbosa, P., Krischik, V. A. & Jones, C. *Microbial Mediation of Plant-Herbivore Interactions* 1st edn. (Wiley, 1991).

[71] Kinkel, L. L. *Microbial Population Dynamics on Leaves*10.1146/annurev.phyto.35.1.327 (1997). 10.1146/annurev.phyto.35.1.327 15012527

[72] Owen, D. *Tropical Butterflies: The Ecology and Behavior of Butterflies in the Tropics with Special Reference to African Species* 1st edn. (Clarendon Press, 1971).

[73] Wilson, P. A., Room, P. M., Zalucki, M. P. & Chakraborty, S. Interaction between *Helicoverpa armigera* and *Colletotrichum gloeosporioides* on the tropical pasture legume stylosanthes scabra. *Austral. J. Agric. Res.*10.1071/AR98095 (2000).

[74] Berrigan, D. & Charnov, E. L. Reaction norms for age and size at maturity in response to temperature: A puzzle for life historians. *Oikos*10.2307/3545787 (1994).

[75] Gotthard, K., Nylin, S. & Nylin, S. Adaptive plasticity and plasticity as an adaptation: A selective review of plasticity in animal morphology and life history. *Oikos*10.2307/3545669 (1995).

[76] Arendt, J. D. Adaptive intrinsic growth rates: An integration across taxa. *Q. Rev. Biol.*10.1086/419764 (1997).

[77] Blanckenhorn, W. U. Different growth responses to temperature and resource limitation in three fly species with similar life histories. *Evol. Ecol.*10.1023/A:1006741222586 (1999).

[78] Tan, Y. Q., Dion, E. & Monteiro, A. Haze smoke impacts survival and development of butterflies. *Sci. Rep.*10.1038/s41598-018-34043-0 (2018). 30353024 10.1038/s41598-018-34043-0 PMC6199247

[79] Rocha-Ortega, M., Rodriguez, P. & Córdoba-Aguilar, A. Geographical, temporal and taxonomic biases in insect gbif data on biodiversity and extinction. *Ecol. Entomol.* **46**, 718–728. 10.1111/een.13027 (2021).

[80] Sánchez-Bayo, F. & Wyckhuys, K. A. Worldwide decline of the entomofauna: A review of its drivers. *Biol. Conserv.* **232**, 8–27. 10.1016/j.biocon.2019.01.020 (2019).

[81] Collen, B., Ram, M., Zamin, T. & McRae, L. The tropical biodiversity data gap: Addressing disparity in global monitoring. *Trop. Conserv. Sci.* **1**, 75–88. 10.1177/194008290800100202 (2008).

[82] Myers, N., Mittermeler, R. A., Mittermeler, C. G., da Fonseca, G. A. B. & Kent, J. Biodiversity hotspots for conservation priorities. *Nature* **403**, 853–858. 10.1038/35002501 (2000). 10706275 10.1038/35002501

[83] Gilbert, L. E. *Ecological Consequences of a Coevolved Mutualism Between Butterflies and Plants*, 1st edn. 210–240 (University of Texas Press, 1975).

[84] Team, R. C. *R: A Language and Environment for Statistical Computing* (R Foundation for Statistical Computing, 2021).

[85] Team, Q. D. *QGIS Geographic Information System* (QGIS Association, 2024).

[86] Wickham, H. *ggplot2: Elegant Graphics for Data Analysis* (Springer, 2016).

