# Supplementary material for: Effects of fine particulate matter air pollution on survival of Heliconius ethilla (Godart, 1819)
Source: Sci Rep. 2024 Nov 29;14:29710. doi: 10.1038/s41598-024-78347-w (PMC11606968; doi:10.1038/s41598-024-78347-w)
Supplement: Supplementary file 1 — Supplementary Tables. [file 41598_2024_78347_MOESM1_ESM.pdf]

Supplementary Material

EFFECTS OF FINE PARTICULATE MATTER AIR POLLUTION ON SURVIVAL OF HELICONIUS ETHILLA (GODART, 1819)

March 12, 2024

x	Group	S(x)	l(x)	p(x)	q(x)	L(x)	T(x)	e(x)
Egg	Control	3255	1	0.261	0.739	0.631	1.921	1.921
L1		850	0.261	0.908	0.092	0.249	1.290	4.940
L2		772	0.237	0.948	0.052	0.231	1.041	4.389
L3	Control	732	0.225	0.941	0.059	0.218	0.810	3.601
	Treatment	69	0.739	0.739	0.261	0.652	1.775	2.402
L4	Control	689	0.212	0.961	0.039	0.208	0.592	2.795
	Treatment	51	0.565	0.765	0.235	0.500	1.123	1.987
L5	Control	662	0.203	0.831	0.169	0.186	0.384	1.888
	Treatment	39	0.435	0.769	0.231	0.420	0.623	1.433
Pupa	Control	550	0.169	0.671	0.329	0.141	0.198	1.171
	Treatment	30	0.406	0.933	0.067	0.203	0.203	0.500

Table 1: Life Table of H. ethilla of the Control and Treatment groups in 25 ± 0,3°C and photoperiod of 12 hours (x = stage of life; S(x) = individuals in each instar or age class; l(x) = probability of survival; p(x) = probability of survival to the next stage; q(x) = probability of death; L(x) = proportion of the population that lived until stage x; T(x) = proportion of the population that lived until stage x and in all subsequent stages; e(x) = life expectancy.

Stage	Group	H ± SD*	95% CI (Lower)	95% CI (Upper)
Egg		3.95 ± 1.04	3,91	3,99
L1	Control	2.31 ± 0.68	2,29	2,33
L2		2.38 ± 0.98	2,35	2,41
	Control	2.75 ± 1	2,72	2,78
L3	Treatment	3.40 ± 0.95	3,18	3,62
	Control	3.17 ± 1.02	3,13	3,21
L4	Treatment	3.82 ± 1.05	3,57	4,07
	Control	4.44 ± 1.10	4,40	4,48
L5	Treatment	5.55 ± 1.19	5,27	5,83
	Control	10.27 ± 1.09	10,23	10,31
Pupa	Treatment	11.64 ± 1.15	11,37	11,91
	Control	29.27 ± 2.17	29,20	29,34
Complete Cycle	Treatment	33.59 ± 3.17	32,84	34,34

Table 2: Time of development in days (H) of the life cycle of *Heliconius ethilla* in each stage from the Control and Treatment groups in 25 ± 0.3°C and photoperiod of 12 hours. *SD = Standard Deviation and CI = Confidence Interval, with the lower and upper 95% Confidence Intervals.
